# Prognostic impact of HER2 biomarker levels in trastuzumab-treated early HER2-positive breast cancer

**DOI:** 10.1186/s13058-024-01779-9

**Published:** 2024-02-07

**Authors:** Caroline Rönnlund, Emmanouil G. Sifakis, Caroline Schagerholm, Qiao Yang, Emelie Karlsson, Xinsong Chen, Theodoros Foukakis, Jodi Weidler, Michael Bates, Irma Fredriksson, Stephanie Robertson, Johan Hartman

**Affiliations:** 1 Department of Oncology and Pathology, Karolinska Institutet, Visionsgatan 56, CCK R8:04, 17176 Stockholm, Sweden; 2 Department of Clinical Pathology and Cancer Diagnostics, Karolinska University Hospital, Stockholm, Sweden; 3 Breast Center, Theme Cancer, Karolinska University Hospital, Stockholm, Sweden; 4 Medical and Scientific Affairs and Strategy, Oncology, Cepheid, Sunnyvale, CA USA; 5 Department of Molecular Medicine and Surgery, Karolinska Institutet, Stockholm, Sweden; 6 Department of Breast-, Endocrine Tumors and Sarcoma, Karolinska University Hospital, Stockholm, Sweden; 7 Medtechlabs, Bioclinicum, Karolinska University Hospital, Stockholm, Sweden

**Keywords:** Breast cancer, HER2, *ERBB2* mRNA, Digital image analysis, Prognosis

## Abstract

**Background:**

Overexpression of human epidermal growth factor receptor 2 (HER2) caused by HER2 gene amplification is a driver in breast cancer tumorigenesis. We aimed to investigate the prognostic significance of manual scoring and digital image analysis (DIA) algorithm assessment of HER2 copy numbers and HER2/CEP17 ratios, along with *ERBB2* mRNA levels among early-stage HER2-positive breast cancer patients treated with trastuzumab.

**Methods:**

This retrospective study comprised 371 early HER2-positive breast cancer patients treated with adjuvant trastuzumab, with HER2 re-testing performed on whole tumor sections. Digitized tumor tissue slides were manually scored and assessed with uPath HER2 Dual ISH image analysis, breast algorithm. Targeted *ERBB2* mRNA levels were assessed by the Xpert® Breast Cancer STRAT4 Assay. HER2 copy number and HER2/CEP17 ratio from in situ hybridization assessment, along with *ERBB2* mRNA levels, were explored in relation to recurrence-free survival (RFS).

**Results:**

The analysis showed that patients with tumors with the highest and lowest manually counted HER2 copy number levels had worse RFS than those with intermediate levels (HR = 2.7, CI 1.4–5.3, *p* = 0.003 and HR = 2.1, CI 1.1–3.9, *p* = 0.03, respectively). A similar trend was observed for HER2/CEP17 ratio, and the DIA algorithm confirmed the results. Moreover, patients with tumors with the highest and the lowest values of *ERBB2* mRNA had a significantly worse prognosis (HR = 2.7, CI 1.4–5.1, *p* = 0.003 and HR = 2.8, CI 1.4–5.5, *p* = 0.004, respectively) compared to those with intermediate levels.

**Conclusions:**

Our findings suggest that the association between any of the three HER2 biomarkers and RFS was nonlinear. Patients with tumors with the highest levels of HER2 gene amplification or *ERBB2* mRNA were associated with a worse prognosis than those with intermediate levels, which is of importance to investigate in future clinical trials studying HER2-targeted therapy.

**Supplementary Information:**

The online version contains supplementary material available at 10.1186/s13058-024-01779-9.

## Background

Human epidermal growth factor receptor 2 (HER2) is encoded by the oncogene *ERBB2* and is overexpressed in around 15% of all primary breast cancers [1, 2]. In clinical routine, HER2 status is determined with HER2 protein expression analyzed by immunohistochemistry (IHC) and HER2 gene amplification assessed by a DNA probe integrated into an in situ hybridization (ISH) detection system. HER2 was discovered decades ago, but the same diagnostic methods are still in use despite being time-consuming and hampering reproducibility problems [2–4]. Several other techniques for determining HER2 status at either protein, RNA or DNA levels are available but have not yet reached routine practice [5]. In addition, HER2-positive breast cancer patients are treated similarly despite individual variations in HER2 copy number and HER2/Chromosome enumeration probe 17 (CEP17) ratio levels.

Unlike HER2 diagnostic methods, treatment options for HER2-positive breast cancer patients have evolved rapidly over the last decades [6–11]. Today, high-risk early-stage HER2-positive breast cancer patients are most often recommended neoadjuvant treatment with a combination of monoclonal antibodies that bind to the extracellular domain of the HER2 receptor (e.g., trastuzumab and pertuzumab) together with chemotherapy [3, 11–13]. Post-surgery, patients usually receive additional trastuzumab, but in case of residual disease, the antibody–drug conjugate (ADC) trastuzumab emtansine (T-DM1), which combines trastuzumab with a cytotoxic drug called emtansine or DM1 is recommended [3, 7, 14]. Recently, new, more effective ADCs for HER2-positive breast cancer, such as trastuzumab deruxtecan (T-DXd), have been introduced [15, 16]. In addition, T-DXd demonstrated activity against HER2 low tumors (i.e., HER2 IHC 1+ and non-amplified HER2 IHC 2+) [17]. In the era of new-generation ADCs, it is essential to improve the definition of HER2 testing. Therefore, there is an interest in investigating HER2 biomarker levels in relation to outcomes among HER2-positive breast cancer. In addition, local and regional variations in HER2 positivity rates are evident, and it is essential to limit false-negative and false-positive HER2 results through standardization and quality control [18–20].

One solution to standardize HER2 assessment and reduce HER2 analysis time is through digital image analysis (DIA). Current studies mainly focus on HER2 IHC scoring [21–23]. Apart from a few artificial intelligence (AI)-based models that have been developed to perform IHC scoring from IHC-stained slides, another model has demonstrated the capability to predict HER2 status directly from hematoxylin and eosin (H&E)-stained slides [24, 25]. Recently, an ISH digital image analysis algorithm that could aid pathologists in investigating HER2 at a DNA level was developed [26–28]. Since ISH scoring is the most time-consuming procedure in HER2 diagnostics and is hampered by variability between readers, decision aids are essential for pathologists and patients.

This study aimed to systematically re-assess HER2 biomarkers including HER2 ISH and *ERBB2* mRNA, and correlate them to outcomes in trastuzumab-treated early HER2-positive breast cancer patients. We aimed to investigate continuous levels of the biomarkers and their prognostic potential to discriminate between patients with therapy-resistant disease and those with good outcomes.

## Materials and methods

### Stockholm HER2 cohort study design

**Fig. 1:**
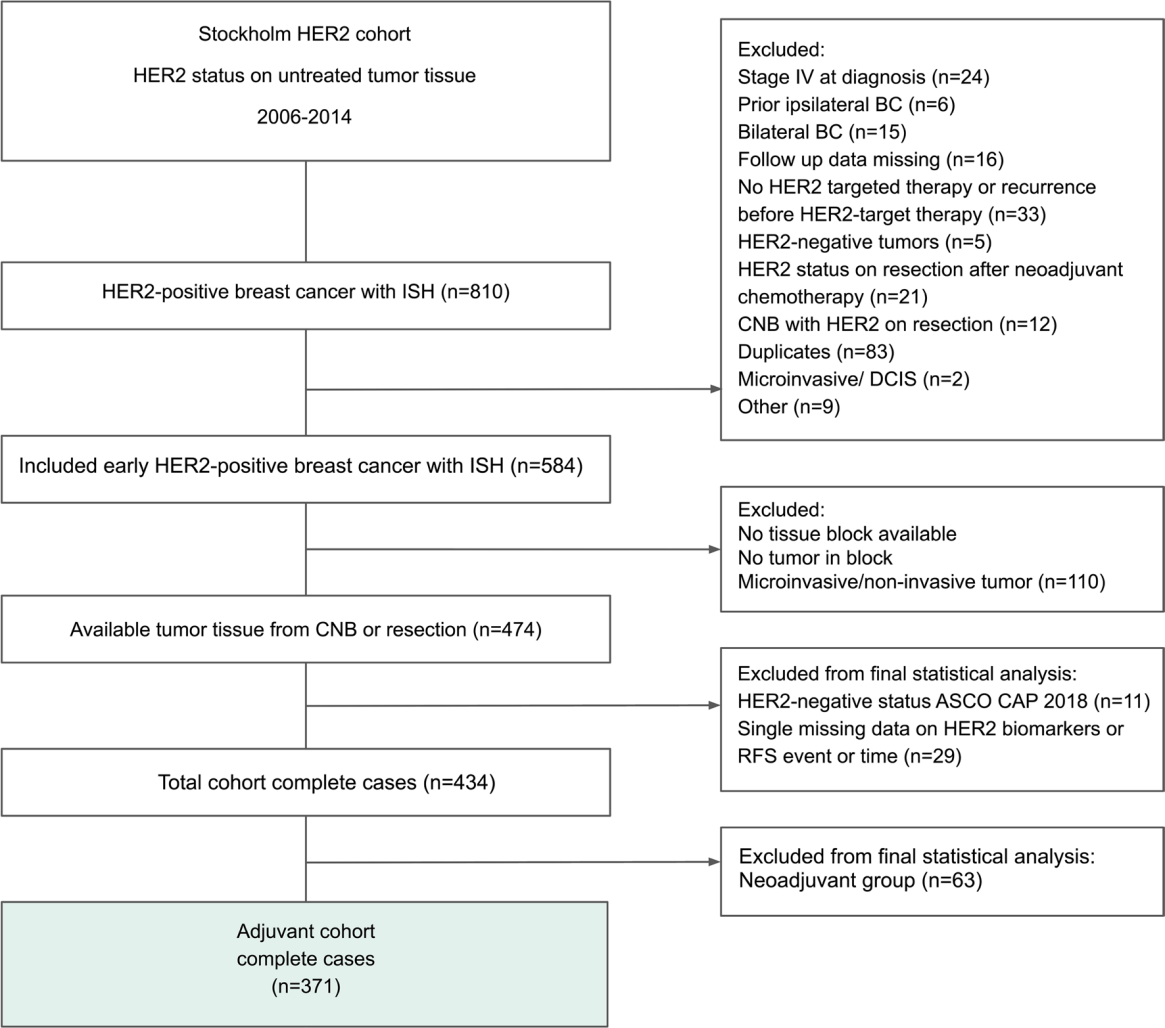
CONSORT diagram of the Stockholm HER2 cohort. *ASCO* American Society of Clinical Oncology, *BC* breast cancer, *CAP* College of American Pathologists, *CNB* core needle biopsy, *DCIS* ductal cancer in situ, *ISH* in situ hybridization, *RFS* recurrence-free survival

The Stockholm HER2 cohort is a retrospective identified cohort comprising patients diagnosed with HER2-positive early-stage primary breast cancer at the Department of Clinical Pathology and Cancer Diagnostics at the Karolinska University Hospital, Stockholm, Sweden, between 2006 and 2014. The pathology laboratory information system was searched to identify all HER2-analyzed tumors at the Karolinska University Hospital and to extract clinicopathological data (tumor size, axillary lymph node status, estrogen receptor (ER), progesterone receptor (PR), Ki67, HER2 by IHC and ISH). In addition, treatment information and at least 5-year follow-up data were extracted from medical records. The outcome variable was recurrence-free survival (RFS), defined as the time from the pathology-verified diagnosis until the time of recurrence or death by any cause, according to the STEEP criteria [29]. Exclusion criteria were as follows: HER2-negative breast cancer, previous ipsilateral breast cancer diagnosis, bilateral breast cancer, stage IV disease at diagnosis, recurrence before HER2-targeted therapy, no HER2-targeted treatment, lack of follow-up data, no HER2 status on untreated tumor tissue, no invasive tumor or duplicates (Fig. 1).

After exclusions, the study cohort comprised 474 primary tumors with available original HER2 status analyzed on either core needle biopsy or surgical resection specimen prior to treatment. Archived tumor tissue material from the Stockholm medical biobank was retrieved, sectioned and stained with H&E; stained and analyzed for HER2 IHC and HER2 ISH; and analyzed for *ERBB2* mRNA using Xpert Breast Cancer STRAT4 (CE-IVD. In vitro diagnostic medical device. Not available in all countries. Not available in the U.S) assay. After further exclusion of patients with HER2 negative tumors on re-assessment with the ASCO guidelines updated 2018 [4] and those with incomplete data on one of HER2 gene amplification, *ERBB2* mRNA or outcome data (RFS), a total of 434 patients were available for statistical analysis. Patients had been treated with trastuzumab either in the neoadjuvant setting (14.5%) or the adjuvant setting (85.5%). In this study, we focused on the adjuvant-treated part of the cohort, thus including 371 primary tumors for analysis (Fig. 1). This study was performed and reported to the greatest extent in accordance with the REMARK guidelines [30]. The biospecimen reporting for improved study quality (BRISQ) criteria for this cohort are shown in the Additional file 1: Table S1 [31].

### HER2 immunohistochemistry and in situ hybridization

In the original biomarker assessment, fluorescent or chromogen ISH was routinely performed for all HER2 IHC score 2+  and 3+ tumors. As the fluorescent ISH signals fade over time, all tumors with available tumor tissue were re-tested for HER2 (from December 2019 to June 2020) to accurately compare HER2 status across the cohort. New parallel 4 μm tumor tissue whole sections from the archived formalin-fixed paraffin-embedded (FFPE) material were stained with the PATHWAY anti-HER2/neu (4B5) Rabbit Monoclonal Primary Antibody (Roche Diagnostics International, Rotkreutz, Switzerland) as described by the manufacturer (BenchMark ULTRA IHC/ISH Staining Module, Ventana Medical Systems, Inc., Arizona, USA). Similarly, ISH for HER2 was performed on a parallel 4 μm tumor tissue section. HER2 dual-probe ISH staining utilized VENTANA HER2 Dual ISH DNA Probe Cocktail assay (Roche Diagnostics International, Rotkreutz, Switzerland) together with VENTANA Silver ISH DNP Detection kit and VENTANA Red ISH DIG detection kit as described by the manufacturer (BenchMark ULTRA IHC/ISH Staining Module, Ventana Medical Systems, Inc., Arizona, USA). For accurate histopathological assessment of HER2 status, all tumors also included a parallel whole tumor section stained with H&E.

### Manual HER2 scoring

All new tumor slides (including H&E, HER2 IHC and HER2 ISH) were scanned at 40X using a NanoZoomer XR (Hamamatsu Photonics K.K., Japan) digital slide scanner. The NDP.view2 (Hamamatsu Photonics K.K., Japan) viewing software was used to view the whole slide images. All re-tested HER2 sections were evaluated by at least two pathologists (CR resident pathologist and JH or SR, both board-certified breast pathologists). The manual scoring of IHC and ISH was performed at 40-60X on digitized whole slide images or by brightfield light microscopy for a few cases. According to the Swedish national guidelines, the number of HER2 signals and CEP17 signals per cell was counted in 20 tumor cells in two separate areas of the invasive tumor. The average HER2 signals/cell (hereafter copy number) and HER2/CEP17 ratio were reported for each case. HER2 IHC score 0 to 3+ was defined as follows: 0 as no or incomplete faint/weak membrane staining in ≤ 10%; 1+ as incomplete faint/weak membrane staining in > 10%; 2+ as weak to moderate complete membrane staining in > 10%, or complete intense staining in ≤ 10%; 3+ as complete intense membrane staining in > 10% of tumor cells [4, 32, 33]. IHC 0–1+ was defined as negative, IHC 2+ as equivocal requiring reflex test with ISH, and IHC 3+ as positive [33].

The Swedish national guidelines of 2020 were used for HER2 re-testing and are in line with the ASCO/CAP 2018 guidelines [4, 32, 33]; tumors assessed as IHC 3+ or 2+ together with a HER2/CEP17 ratio ≥ 2 with HER2 copy number ≥ 4 signals/cell (ASCO/CAP dual ISH group 1) or HER2/CEP17 ratio < 2 with HER2 copy number ≥ 6 signals/cell (ISH group 3) were considered as HER2-positive. Thus, ISH group 5 was considered negative (HER2/CEP17 ratio < 2.0 and HER2 copy number < 4.0 signals/cell). The ASCO/CAP dual ISH groups 2 and 4 are the less common clinical scenarios and were assessed according to the ASCO CAP algorithm [32].

### HER2 scoring by digital image analysis

All tumor slides were scanned at 40X for ISH using the VENTANA DP 200 slide scanner (Roche Diagnostics International, Rotkreuz, Switzerland). The images were analyzed with the uPath HER2 Dual ISH image analysis, breast algorithm (Roche Diagnostics International, Rotkreuz, Switzerland) between June 2022 to December 2022 using Roche uPath enterprise software (Roche Diagnostics International, Rotkreuz, Switzerland). The DIA algorithm scoring was performed (by CR) with a substantial wash-out period of at least 18 months from the manual scoring. uPath HER2 Dual ISH image analysis, breast algorithm is a partly automatic DIA algorithm that imitates a pathologist’s ISH assessment and has been described previously [26]. Briefly, the pathologist identified two regions of interest (ROIs) using the provided heatmaps within the viewer and excluded non-invasive areas and areas with low HER2 amplification. Thereafter, the DIA algorithm selected 20 cells per ROI and automatically output each cell's HER2 copy number count and a CEP17 count. Finally, the pathologist confirmed each cell count and if needed, deleted unacceptable cells and chose new countable tumor cells. Eventually, the DIA algorithm summarized all counted cells and presented an average HER2 copy number and HER2/CEP17 ratio per analyzed case. The HER2 scoring by the DIA algorithm was performed and reported separately from the manual HER2 scoring results, *ERBB2* mRNA results and the outcome endpoint.

### *ERBB2* mRNA by RT-PCR

To measure *ERBB2* mRNA, the CE-IVD marked (In vitro diagnostic medical device. Not available in all countries. Not available in the U.S.) analysis Xpert® Breast Cancer STRAT4 Assay (Cepheid, Sunnyvale, CA, USA) based on real-time polymerase chain reaction (RT-PCR) was performed between December 2019 and June 2020. From archived FFPE material, macrodissection (with > 90% tumor content) of each invasive tumor was performed and a 10 μm tumor tissue section cut and placed into tubes. The tumor material was prepared according to the manufacturer’s instructions (Xpert Breast Cancer STRAT4® Assay, Cepheid, Sunnyvale, CA, USA) [34]. Briefly, the samples were treated with FFPE lysis reagent and Proteinase K and incubated at 80 °C for 30 min. Next, the content was diluted with ≥ 95% ethanol, and the lysate was added to the closed-system STRAT4® cartridge and analyzed at the semi-automatic GeneXpert (GX system) which performed isolation of RNA, amplification and real-time detection of mRNA. Cycle thresholds (Ct) were determined for *ERBB2* and the endogenous reference gene called Cytoplasmic FMR1-Interacting Protein 1 (*CYFIP1).* The *ERBB2* mRNA results were presented as a delta Ct (dCt) value, which was defined as the *CYFIP1* Ct minus the *ERBB2* Ct. ≥ − 1.0 dCt was the predetermined cutoff referred to as an *ERBB2* mRNA positive result. The cutoff was provided by the manufacturer and was based on previous studies [34–36]. The *ERBB2* mRNA analysis was performed blinded from the manual HER2 assessment and the DIA algorithm scoring.

### Statistical analysis

Methods to search for optimal cutoffs were applied for the study cohort *n* = 371 according to Fig. 1, and patients were classified as low, intermediate or high for each investigated biomarker. Specifically, cutoff determination for HER2 copy number (manual and DIA algorithm), HER2/CEP17 ratio (manual and DIA algorithm) and *ERBB2* mRNA was investigated by positional scanning (PS) analysis using Cutoff Finder [37]. Cutoff Finder selects the optimal cutoff associated with the lowest p-value for RFS. In addition, subpopulation treatment effect pattern plot (STEPP) analysis suggested a second cutoff, which was also defined by positional scanning as described previously [38–41]. For the STEPP analysis, which examines the relationship between a continuous covariate and the probability of survival at a predefined time point, a sliding-window approach was used to define several overlapping subpopulations of patients [41]. The optimal values for generating the patient subpopulations were defined by the utility function *balance_patients* in R package *stepp*. (This divided the cohort into 9–11 subgroups, with approximately 33–41 patients in each group, depending on the biomarker in question.) The reverse Kaplan–Meier (KM) estimate of the median RFS follow-up was used as the predefined time point in the STEPP analysis [42].

The low, intermediate and high groups of patients were used for survival analysis, which was performed with the R package *survival* [43] using RFS as the clinical endpoint. Specifically, KM estimates and univariate and multivariable Cox proportional hazards (PH) regression models were applied, and hazard ratios (HRs) with 95% confidence intervals (CIs) were estimated. The PH assumption was checked with the scaled Schoenfeld residuals. Continuous co-variables in the multivariable Cox regression models were tested for nonlinearity by plotting the Martingale residuals. Estrogen receptor status was found to violate the PH assumption, and thus, it was used as a stratification factor in the multivariable Cox regression models, which also included tumor size (categorical, ≤ 20 mm; > 20 to ≤ 50 mm; > 50 mm) and lymph node status (categorical, LN−; LN+). All statistical tests were two-sided. Significance was considered at a *p* < 0.05 level. All bioinformatics and statistical analyses including descriptive tumor characteristics were performed within the R computing environment version 4.2.2 (2022-10-31) or later, © 2022 by Posit Software, PBC.

## Results

### Patient characteristics

Results showed that 327 (88.1%) of the patients had tumors that were IHC 3+, 42 (11.3%) were IHC 2+ and 2 (0.5%) were IHC 1+ . The median average HER2 signals/cell by manual scoring was 9.8 signals/cell (range 2.3–21.6), and the median HER2/CEP17 ratio by manual scoring was 5.8 (range 1.4–18.8). Among all tumors, 226 (60.9%) were ER-positive (cutoff ≥ 10%), 140 (37.7%) were PR-positive (cutoff ≥ 10%) and 322 (86.8%) had high Ki67 (cutoff ≥ 20%). All patients were treated with adjuvant trastuzumab and 360 (97.0%) were treated with chemotherapy. During follow-up, 38 (10.2%) of the patients experienced a recurrence of which a majority 30 (78.9%) had a distant metastasis as the first recurrence. The median RFS time was 8.7 years (defined by the reverse Kaplan–Meier estimate) [42]. Complete tumor characteristics, treatment and outcome data of the study cohort (*n* = 371) are presented in the Additional file 1: Tables S2-5.

### Prognostic levels of HER2 copy number and HER2/CEP17 ratio

**Fig. 2:**
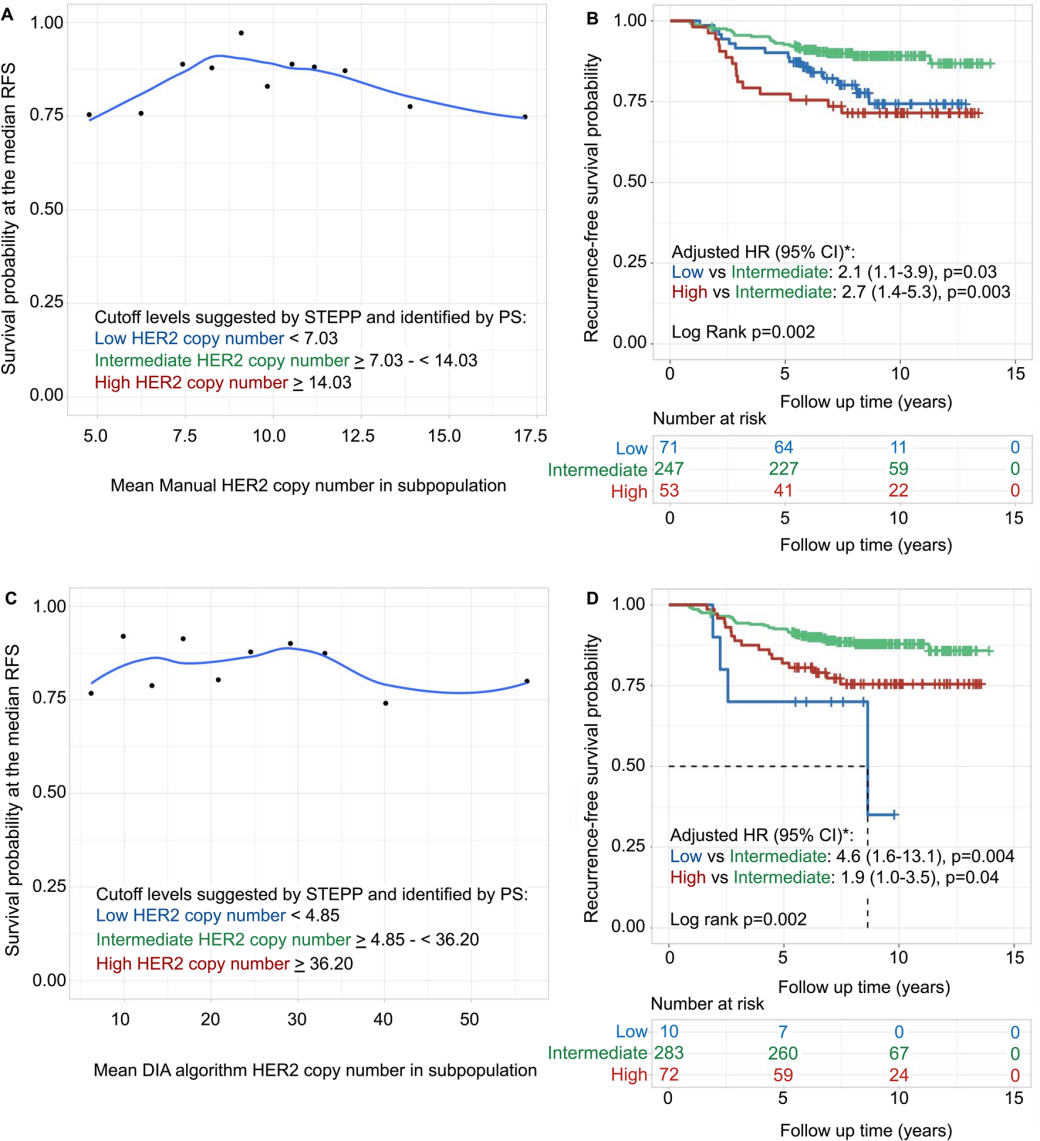
HER2 copy number investigated with subpopulation treatment effect pattern plot (STEPP) and survival analysis. STEPP investigated the relationship between manually- (**A**) and digital image analysis (DIA) algorithm-scored (**C**) mean HER2 copy number and the probability of being recurrence-free at the median recurrence-free survival (RFS) (8.7 years). The lower left corner in **A**, **C** shows the cutoff levels suggested by STEPP analysis and identified by positional scanning (PS). These cutoffs divided the cohort into three subgroups, namely low, intermediate and high. The locally estimated scatterplot smoothing curve fit is shown with the blue line. Kaplan–Meier estimates and multivariable Cox regression model of manually- (**B**) and DIA algorithm-scored (**D**) HER2 copy number related to RFS. The study cohort was divided into three subgroups according to the suggested optimal first and second cutoffs for the manually scored HER2 copy number and the DIA algorithm scored HER2 copy number as shown in **A**, **C**. *The multivariable Cox regression model was adjusted for lymph node status, tumor size and stratified by estrogen receptor status. Adjusted hazard ratios (HR) with 95% confidence interval (CI) are presented

**Fig. 3:**
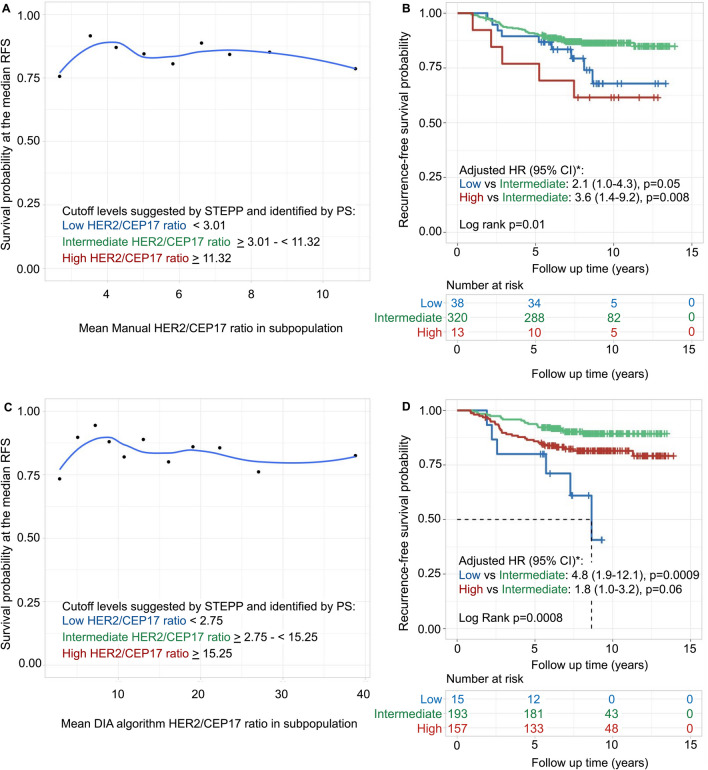
HER2/CEP17 ratio investigated with subpopulation treatment effect pattern plot (STEPP) and survival analysis. STEPP investigated the relationship between manually- (**A**) and digital image analysis (DIA) algorithm-scored (**C**) mean HER2/CEP17 ratio and the probability of being recurrence-free at the median recurrence-free survival (RFS) (8.7 years). The lower left corners in **A**, **C** show the cutoff levels suggested by STEPP analysis and identified by positional scanning (PS). These cutoffs divided the cohort into three subgroups, namely low, intermediate and high. The locally estimated scatterplot smoothing curve fit is shown with the blue line. Kaplan–Meier estimates and multivariable Cox regression model of manually- (**B**) and DIA algorithm-scored (**D**) HER2/CEP17 ratio related to RFS. The study cohort was divided into three subgroups according to the suggested optimal first and second cutoffs for the manually scored HER2/CEP17 ratio and the DIA algorithm scored HER2/CEP17 ratio as shown in **A**, **C**. *The multivariable Cox regression model for manual HER2/CEP17 ratio was adjusted for lymph node status, tumor size and stratified for estrogen receptor status. Adjusted hazard ratios (HR) with 95% confidence interval (CI) are presented

To determine the prognostic significance of different levels of manual and DIA algorithm scoring of HER2 gene amplification, positional scanning with Cutoff Finder along with STEPP analysis were performed and presented in Figs. 2 and 3.

For the manually scored HER2 copy number, cutoffs were suggested at 14.03 and 7.03 signals/cell in relation to RFS (log-rank *p* = 0.002), and the STEPP curve indicated that patients with tumors with intermediate levels of HER2 copy number were associated with superior outcomes (Figs. 2A, B and Additional file 1: Table S6). In multivariable Cox regression analysis, patients with tumors with high levels of HER2 copy number (≥ 14.03 signals/cell) showed a significantly worse RFS than patients with tumors with intermediate levels (HR = 2.7, CI 1.4–5.3, *p* = 0.003). In addition, the patients with tumors with the lowest levels of HER2 copy number (< 7.03 signals/cell) were associated with a significantly worse outcome than those with intermediate levels (HR = 2.1, CI 1.1–3.9, *p* = 0.03; Fig. 2B). The DIA algorithm estimation of HER2 copy number was higher than the manual scoring with a median DIA algorithm HER2 copy number of 22.3 signals/cell compared to the median manual HER2 copy number of 9.8 signals/cell. DIA algorithm showed just as manual counting, although with different cutoffs, that patients with tumors with intermediate HER2 copy numbers were associated with a significantly better prognosis than those with both high levels of HER2 copy number (HR = 1.9, CI 1.0–3.5, *p* = 0.04) and low levels (HR = 4.6, CI 1.6–13.1, *p* = 0.004; Fig. 2C, D).

For the manually scored HER2/CEP17 ratio, cutoffs were suggested at 11.32 and 3.01 in relation to RFS (log-rank *p* = 0.01), indicating that patients with tumors with both high and low ratio levels were associated with a worse prognosis than those with intermediate levels (Fig. 3A, B and Additional file 1: Table S6). Similarly, as the HER2 copy number levels showed, patients with tumors with the highest levels of HER2/CEP17 ratio (≥ 11.32) were associated with a worse prognosis when compared to intermediate levels (HR = 3.6, CI 1.4–9.2, *p* = 0.008), and for the patients with tumors with the lowest levels of HER2/CEP17 ratio (< 3.01) there was a trend for the worse outcome when compared to intermediate levels (HR = 2.1, CI 1.0–4.3, *p* = 0.05; Fig. 3B).

DIA algorithm scoring of HER2/CEP17 ratio confirmed the findings from the manual scoring but with different cutoffs. Patients with tumors with high HER2/CEP17 ratio levels showed a trend toward worse prognosis as compared to those with intermediate levels (HR = 1.8, CI 1.0–3.2, *p* = 0.06) and patients with tumors with low levels of HER2/CEP17 ratio had a significantly worse prognosis than those with intermediate levels (HR = 4.8, CI 1.9–12.1, *p* = 0.0009), (Fig. 3C, D). The HER2 DIA algorithm failed to scan or analyze six tumor slides. In addition, for each case with two ROIs (40 cells), a median of 3.0 (range 0–29) cells was changed by the resident pathologist (CR) because only parts of the cell nuclei were annotated as cells or non-tumor cells were counted.

### Prognostic levels of *ERBB2* mRNA

**Fig. 4:**
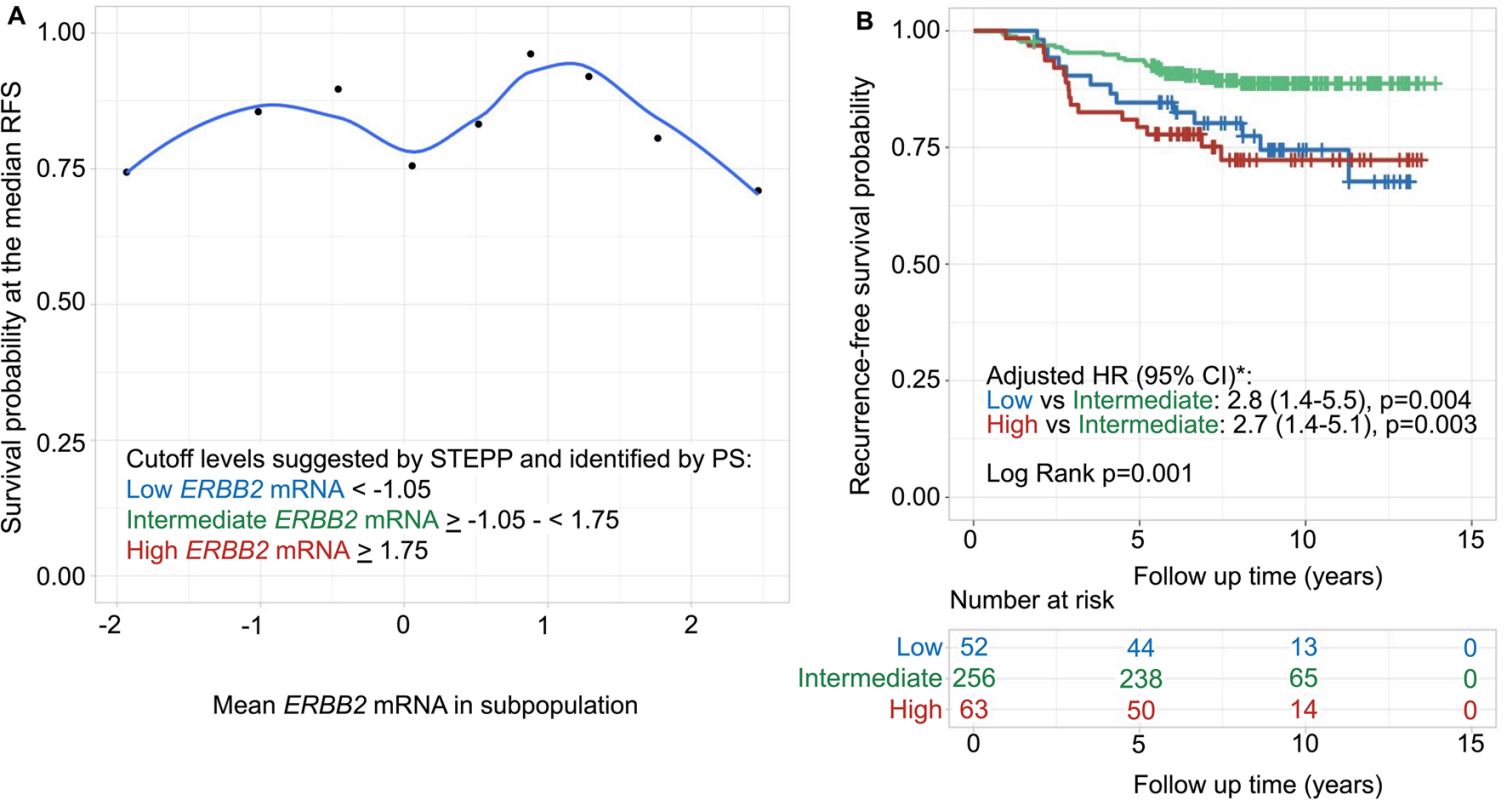
*ERBB2* mRNA investigated with subpopulation treatment effect pattern plot (STEPP) and survival analysis. STEPP investigated the relationship between mean *ERBB2* mRNA (**A**) and the probability of being recurrence-free at the median recurrence-free survival (RFS) (8.7 years). The lower left corner shows the cutoff levels suggested by STEPP analysis and identified by positional scanning (PS). These cutoffs divided the cohort into three subgroups, namely low, intermediate and high. The locally estimated scatterplot smoothing curve fit is shown with the blue line. Kaplan–Meier estimates and multivariable Cox regression model of *ERBB2* mRNA related to RFS (**B**). The study cohort was divided into three subgroups according to the suggested optimal first and second cutoffs for *ERBB2* mRNA as shown in **A**. *The multivariable Cox regression model was adjusted for lymph node status, tumor size and stratified by estrogen receptor status. Adjusted hazard ratios (HR) with 95% confidence interval (CI) are presented

For STRAT4 *ERBB2* mRNA positional scanning with Cutoff Finder along with STEPP analysis suggested cutoffs at 1.75 dCt and − 1.05 dCt in relation to RFS (log rank *p* = 0.001). Notably, the second cutoff was similar to the manufacturer’s set cutoff at − 1.0 dCt. The STEPP curve confirmed the previous pattern observed for HER2 copy number and HER2/CEP17 ratio, and patients with tumors with intermediate levels of *ERBB2* mRNA were associated with significantly better survival outcomes (Fig. 4 and Additional file 1: Table S6). Patients with tumors with the highest *ERBB2* mRNA levels (≥ 1.75 dCt) and lowest *ERBB2* mRNA (< − 1.05 dCt) had a substantially worse RFS than those with tumors showing intermediate levels (HR = 2.7, CI 1.4–5.1, *p* = 0.003 and HR = 2.8, CI 1.4–5.5, *p* = 0.004, respectively).

## Discussion

Recognizing the importance of precise levels of HER2 quantification is paramount with the emergence of novel treatments targeting HER2 in breast cancer. This understanding is crucial for including suitable candidates for clinical trials and, in the long run, for clinical utility. In this study, we systematically re-assessed HER2 copy number, HER2/CEP17 ratio and *ERBB2* mRNA in a trastuzumab-treated breast cancer cohort and investigated the biomarkers in a continuous manner in relation to RFS. We showed that patients with tumors with intermediate levels of HER2 copy number, HER2/CEP17 ratio and *ERBB2* mRNA had a superior prognosis compared to patients with tumors with both high and low levels of each biomarker. Furthermore, we confirmed the findings from manual scoring of HER2 gene amplification levels with a DIA algorithm scoring method.

Surprisingly, in this study none of the three HER2 biomarkers investigated by PS and STEPP analysis were linearly associated with outcome. Instead, we demonstrated that patients with tumors with the highest levels of HER2 copy number, HER2/CEP17 ratio and *ERBB2* mRNA were consistently associated with worse RFS than those with intermediate levels of each biomarker. A few earlier studies have reported similar findings for individual HER2 biomarkers; STEPP analysis of HER2 copy number in the APHINITY trial showed that the lymph node-positive patients in the pertuzumab–trastuzumab treated group with tumors with the highest and the lowest levels of HER2 copy number had a lower treatment effect than those with intermediate levels of HER2 copy number [44]. Moreover, PS and STEPP analysis were used in two additional studies, one in the adjuvant and one in the metastatic setting. Both investigated HER2 at the protein level with the HERmark® assay (Monogram Biosciences Inc., South San Francisco, CA, USA) and found that patients treated with trastuzumab with very high tumor HER2 expression were associated with worse prognosis [38, 45]. In the present study, we investigated continuous assessments of HER2 in tumors at both the DNA and RNA levels, which to some extent, provides an internal validation of our findings. Moreover, to our knowledge, continuous assessments of *ERBB2* mRNA by STEPP have not been published previously.

The recently published DAISY trial investigating the efficacy of T-DXd in metastatic breast cancer patients with variable HER2 expression has increased the interest in presenting detailed information on HER2 biomarker levels in clinical trials, and stakes have been raised to improve the speed of HER2 assessment in routine pathology [46].

Even though the DAISY trial enrolled patients based on HER2 IHC assessments and did not focus on detailed information on HER2 copy number and HER2 CEP/17 ratios, it may be interesting to investigate HER2 amplification levels in future studies since the ISH analysis is essential for clinical treatment decisions in HER2-positive breast cancer. However, ISH is a time-consuming procedure, and an automated digital assessment tool for ISH could provide valuable support. In the present study, the prognostic value of the DIA algorithm was similar to manual scoring.

As previously discussed by Griguolo et al. [47], cutoff levels for ADC treatment effect may differ depending on the type of molecule, indicating the importance of searching for relevant cutoff levels as in our study. For instance, Perez et al. showed that progression-free survival was superior in the T-DM1 group with higher *ERBB2* mRNA (≥ median) compared to the trastuzumab group [48]. In addition, another trial of metastatic HER2-positive breast cancer confirmed a greater benefit for T-DM1 in contrast to physicians’ choice (typically trastuzumab-based regimes) in patients with tumors with high HER2 expression [49].

This hypothesis aligns with our results, indicating a worse prognosis in trastuzumab-treated HER2-positive patients with the highest HER2 biomarker levels and is also shown elsewhere [48, 50–52]. However, results have varied, mainly in the neoadjuvant setting, where high levels of HER2 gene amplification or HER2 gene expression have been associated with pathological complete response [53–56]. Subgroup analysis of adjuvant trastuzumab-treated HER2-positive patients showed a trend for less benefit in patients with the highest levels of HER2 copy number, but the results were insignificant [57].

In patients with residual disease after neoadjuvant HER2 target treatment, gene expression analysis of biomarkers revealed that high *ERBB2* mRNA expression (> median) was associated with worse invasive disease-free survival within the group treated with trastuzumab in contrast to the T-DM1 group [50]. Considering our findings, we speculate that trastuzumab-treated patients with the highest biomarker levels may benefit from additional or more effective HER2 target treatment, e.g., ADCs.

Explanations as to why patients with tumors showing the highest and the lowest HER2 copy number, HER2/CEP17 ratio and *ERBB2* mRNA had worse prognosis could possibly be found in the mechanistic investigations of HER2 therapy resistance, which are outside of the scope of this study and are therefore briefly described. Resistance to trastuzumab may occur in HER2-positive breast tumors because of HER2 molecular changes, including impaired or changed HER2 receptor epitope [58–60]. Trastuzumab may also be insufficient in blocking the heterodimerization of other HER family members, such as HER3, which is a resistance mechanism that could be overcome by adding the monoclonal antibody pertuzumab that binds to another part of the extracellular domain of HER2 [61, 62]. Other described causes of trastuzumab resistance are altered or alternative HER2 downstream signaling pathways, changed immune-related processes, changed metabolic processes, tumor cell plasticity, altered angiogenesis or intratumoral heterogeneity [63–69]. Interestingly, in vitro studies have shown that tumor cells with high expression of HER2 had an effective transport of the receptor back to the cell surface compared to cells with low HER2 expression, and one could speculate that this phenomenon might impact trastuzumab efficacy in tumors expressing the highest levels of HER2 [70].

Moreover, HER2-positive tumors with the lowest levels of HER2 gene amplification or HER2 expression may be insensitive to trastuzumab, and intratumor heterogeneity has been shown to impact trastuzumab efficacy [68, 71–73]. Lastly, another simpler explanation might be that patients with tumors with the highest levels of HER2 copy number, HER2/CEP17 ratio and *ERBB2* mRNA could have been underdosed with trastuzumab.

Biological reasons as to why trastuzumab-treated patients with highly expressed or amplified HER2-positive tumors have a worse prognosis than those treated with T-DM1 are probably based on the linked cytotoxic agent of T-DM1 but are not yet thoroughly investigated [74]. It has been shown that T-DM1 keep the biological mechanisms of trastuzumab and that patients who did not have an effect of trastuzumab and taxanes might still respond to T-DM1 [14, 50, 75]. Proposed explanations for differences in response to treatment include downstream signaling alterations, where resistance had been shown for trastuzumab and pertuzumab but not for T-DM1 [76]. In addition, HER2-positive mouse models have demonstrated that T-DM1 treatment enhances T-cell infiltration compared to trastuzumab, indicating immune-related differences [77].

There are certain limitations within our study. Only patients with HER2-positive breast cancer were included in the cohort; no HER2-negative control group existed. *ERBB2* mRNA values may vary depending on the methodology to estimate *ERBB2* mRNA levels, which limits comparisons across studies. RT-PCR and in situ hybridization are different methods to evaluate HER2 in tumor material, and some discordant cases were present; for example, some tumors in the low *ERBB2* mRNA group had intermediate levels of HER2 amplification group and vice versa. A possible explanation for differences might be that there are tumors with heterogenous HER2 amplification or expression.

Positional scanning (by Cutoff Finder) and STEPP analysis are statistical methods based on mathematical estimations. Before results are confirmed in larger cohorts, the presented cutoffs in this study should not be used as an exact cutoff but rather be regarded as estimations of approximate levels of HER2 copy number, HER2/CEP17 ratio and *ERBB2* mRNA where the prognostic value change. The cutoffs differed between DIA algorithm scoring and manual scoring, and this might be explained by the observed difference in the management of HER2 clusters, where DIA algorithm tends to calculate higher HER2 signals in clusters than the manual assessment. The ground truth of cluster estimations would be a DNA sequencing analysis but is outside the scope of this study. Even though the DIA algorithm resulted in different estimations compared to the manual counting, both methods concluded equally that the patients with tumors with the highest and the lowest levels of HER2 gene amplification had a worse prognosis than those with intermediate levels.

Despite limitations, this study presents a unique systematic re-testing of HER2 amplification on whole slide tumor sections. The freshly cut and stained sections provided the best possible quality of slides with the current clinically approved methodology. The reassessments performed by the same resident pathologist and reviewed by one of the two board-certified pathologists blinded to previous HER2 results with the addition of DIA algorithm scoring minimized the risk of variations between observers. DIA algorithm of HER2 copy number and HER2/CEP17 ratio could be a method to standardize assessment and support pathologists’ work, preferably in research where standardization is essential.

## Conclusions

In conclusion, the association between either HER2 gene amplification or *ERBB2* mRNA and outcome appeared nonlinear. Our results consistently showed that trastuzumab-treated HER2-positive patients with tumors with the highest levels of HER2 gene amplification or *ERBB2* mRNA had a worse prognosis than those with intermediate levels. If a future prospective study could show that the patients with tumors with the highest levels of HER2 biomarkers fail on trastuzumab and/or pertuzumab but not T-DXd, this subgroup of patients with the highest levels of HER2 gene amplification or highest levels of *ERBB2* mRNA could be offered T-DXd as an initial treatment for early HER2-positive breast cancer. We propose that evaluating biomarker levels parallel to treatment outcomes will be essential to the study design. A first step in understanding HER2 biomarkers and the risk of recurrence is hereby presented. Further studies are ongoing, including molecular studies to understand the biology behind HER2 resistance.

### Supplementary Information

**Additional file 1**:**Table S1.** BRISQ criteria. **Table S2. **Tumor characteristics of the Stockholm HER2 cohort. **Table S3.** Re-tested HER2 immunohistochemistry and *in situ *hybridization (ISH) results. **Table S4. **Adjuvant treatment data of the Stockholm HER2 cohort. **Table S5.** Outcome data of the Stockholm HER2 cohort. **Table S6.** Suggested biomarker groups by Cutoff Finder and STEPP functions.

## Data Availability

The datasets analyzed during the current study are not publicly available due to local privacy laws but could be available from the corresponding author on reasonable request and after required permissions from the institution and the research collaborators.

## Abbreviations

ADC: Antibody–drug conjugate
AI: Artificial intelligence
BRISQ: Biospecimen reporting for improved study quality
CEP17: Chromosome enumeration probe 17
CI: Confidence intervals
CYFIP1: Cytoplasmic FMR1-Interacting Protein 1
DIA: Digital image analysis
ER: Estrogen receptor
FFPE: Formalin-fixed paraffin-embedded
H&E: Hematoxylin and eosin
HER2: Human epidermal growth factor receptor 2
HR: Hazard ratio
IHC: Immunohistochemistry
ISH: In situ hybridization
KM: Kaplan–Meier
PR: Progesterone receptor
PS: Positional scanning
RFS: Recurrence-free survival
STEPP: Subpopulation treatment effect pattern plot
T-DM1: Trastuzumab emtansine
T-DXd: Trastuzumab deruxtecan
