# Supplementary material for: Prognostic impact of HER2 biomarker levels in trastuzumab-treated early HER2-positive breast cancer
Source: Breast Cancer Res. 2024 Feb 7;26:24. doi: 10.1186/s13058-024-01779-9 (PMC10848443; doi:10.1186/s13058-024-01779-9)
Supplement: Supplementary file 1 — Additional file 1:Table S1. BRISQ criteria. Table S2. Tumor characteristics of the Stockholm HER2 cohort. Table S3. Re-tested HER2 immunohistochemistry and in situ hybridization (ISH) results. Table S4. Adjuvant treatment data of the Stockholm HER2 cohort. Table S5. Outcome data of the Stockholm HER2 cohort. Table S6. Suggested biomarker groups by Cutoff Finder and STEPP functions. [file 13058_2024_1779_MOESM1_ESM.docx]

**Supplementary materials for**

**Prognostic impact of HER2 biomarker levels in trastuzumab-treated early HER2-positive breast cancer**

Caroline Rönnlund, Emmanouil G. Sifakis, Caroline Schagerholm, Qiao Yang, Emelie Karlsson, Xinsong Chen, Theodoros Foukakis, Jodi Weidler, Michael Bates, Irma Fredriksson, Stephanie Robertson^*^ and Johan Hartman^*^

^*^Shared last authorship – equal contributions.

**Corresponding author:**Caroline Rönnlund, MD. Address: Department of Oncology and Pathology, Karolinska Institutet, Visionsgatan 56, CCK R8:04 Karolinska University Hospital Solna, 17176 Stockholm, Sweden. Phone: +46 762633076. E-mail: caroline.ronnlund@ki.se

**Contents:**

**Supplementary Tables**

Table S1. BRISQ criteria

Table S2. Tumor characteristics of the Stockholm HER2 cohort.

Table S3. Re-tested HER2 immunohistochemistry and *in situ* hybridization (ISH) results.

Table S4. Adjuvant treatment data of the Stockholm HER2 cohort.

Table S5. Outcome data of the Stockholm HER2 cohort.

Table S6. Suggested biomarker groups by Cutoff Finder and STEPP functions.

| **Table S1. BRISQ criteria** |  |
| --- | --- |
| **Data elements** | **Information** |
| Biospecimen type and disease status | Untreated primary tumor material |
| Anatomical site | Breast |
| Clinical characteristics of patients | Adjuvant treatment with Trastuzumab  +/- Chemotherapy  +/- Hormone therapy |
| Vital state of patients | Alive at the time of tumor excision |
| Clinical diagnosis of patients | Clinically diagnosed as primary breast cancer |
| Pathology diagnosis | Primary invasive breast cancer, HER2-positive |
| Collection mechanism | Surgical specimen and  a few cases core needle biopsy,  formalin fixation |
| Mechanism of stabilization | None, room temperature |
| Long-term preservation | Formalin fixation |
| Constitution of preservation | 10% formalin |
| Storage temperature | Room temperature |
| Storage duration | 5-14 years |
| Shipping temperature | Room temperature |
| Composition, assessment and selection | Primary trastuzumab-treated HER2-positive tumors with  ISH results available see materials and methods. |

| **Table S2. Tumor characteristics of the Stockholm HER2 cohort** | |
| --- | --- |
|  | **n = 371***^1^* |
| **Diagnostic period** | 2006-2014 |
| **Mean age at diagnosis (years)** | 56 (21, 83) |
| **Tumor tissue sample type for HER2 testing** |  |
| Core needle biopsy | 3 (0.8%) |
| Surgical specimen | 368 (99.2%) |
| **Nottingham histological grade** |  |
| NHG 1 | 10 (2.7%) |
| NHG 2 | 99 (26.7%) |
| NHG 3 | 261 (70.4%) |
| Unknown | 1 (0.3%) |
| **Tumor size** |  |
| pT1 ≤ 20 mm | 211 (56.9%) |
| pT2 > 20 - ≤ 50 mm | 149 (40.2%) |
| pT3 > 50 mm | 10 (2.7%) |
| Unknown | 1 (0.3%) |
| **Lymph node status** |  |
| negative | 226 (60.9%) |
| positive | 145 (39.1%) |
| **Anatomic tumor AJCC 8th stage I-IV** |  |
| stage 0 | 0 (0.0%) |
| stage I | 155 (41.8%) |
| stage II | 172 (46.4%) |
| stage III | 43 (11.6%) |
| stage IV | 0 (0.0%) |
| Unknown | 1 (0.3%) |
|  |  |
| **Estrogen receptor (%)** |  |
| < 1 | 128 (34.5%) |
| ≥ 1 - < 10 | 13 (3.5%) |
| ≥ 10 | 226 (60.9%) |
| Unknown | 4 (1.1%) |
| **Estrogen receptor median (range)** | 62.5 (0.0, 100.0) |
| **Progesterone receptor (%)** |  |
| < 1 | 190 (51.2%) |
| ≥ 1 - < 10 | 40 (10.8%) |
| ≥ 10 | 140 (37.7%) |
| Unknown | 1 (0.3%) |
| **Progesterone receptor median (range)** | 0.0 (0.0, 100.0) |
| **Ki67 (%)** |  |
| < 20 | 49 (13.2%) |
| ≥ 20 | 322 (86.8%) |
| **Ki67 median (range)** | 35.0 (0.0, 95.0) |
| **ERBB2 mRNA median (range)** | 0.6 (-4.2, 3.6) |
| *^1^* Mean (Range); n (%); Median (Range) |  |
|  |  |

| **Table S3. Re-tested HER2 immunohistochemistry and in situ hybridization results** |  |
| --- | --- |
| **HER2 IHC result** | **n = 371***^1^* |
| 0 | 0 (0.0%) |
| 1+ | 2 (0.5%)*^2^* |
| 2+ | 42 (11.3%)*^3^* |
| 3+ | 327 (88.1%) |
| **HER2 ISH average signals/cell** | 9.8 (2.3, 21.6) |
| **HER2 ISH HER2/CEP17** **ratio** | 5.8 (1.4, 18.8) |
| **HER2 ISH groups***^4^* |  |
| Group 1: HER2/CEP17 ratio ≥ 2.0 with HER2 ≥ 4.0 signals/cell | 369 (99.5%) |
| Group 2: HER2/CEP17 ratio ≥ 2.0 with HER2 < 4.0 signals/cell | 0 (0.0%) |
| Group 3: HER2/CEP17 ratio < 2.0 with HER2 ≥ 6.0 signals/cell | 0 (0.0%) |
| Group 4: HER2/CEP17 ratio < 2.0 with HER2 ≥ 4.0 and < 6.0 signals/cell | 0 (0.0%) |
| Group 5: HER2/CEP17 ratio < 2.0 with HER2 < 4.0 signals/cell | 2 (0.5%)*^5^* |
| *^1^* n (%); Median (Range) | |
| *^2^* Both patients were in HER2 ISH group 1  *^3^* All patients were in HER2 ISH group 1  *^4^* Adopted from Wolff et al. 2018  *^5^* Both patients were HER2 IHC 3+ | |

| **Table S4. Adjuvant treatment data of the Stockholm HER2 cohort** |  |
| --- | --- |
| **Adjuvant treatment** | **n = 371***^1^* |
| **HER2-targeted therapy (Trastuzumab)** | 371 (100.00%) |
| **Chemotherapy** | 360 (97.0%) |
| **Endocrine therapy** | 230 (62.0%) |
| **Radiotherapy (N/A=3)** | 301 (81.8%) |
| *^1^* n (%) | |

| **Table S5. Outcome data of the Stockholm HER2 cohort** |  |
| --- | --- |
| **Outcome** | **n = 371***^1^* |
| **Recurrence at end follow-up** |  |
| Recurrence | 38 (10.2%) |
| No recurrence | 333 (89.8%) |
| **Recurrence type** |  |
| Locoregional as first recurrence | 8 (21.1%) |
| Distant metastasis as first recurrence | 30 (78.9%) |
| **Distant metastasis type** |  |
| Distant metastasis lymph node | 2 (6.7%) |
| Distant metastasis organ | 27 (90.0%) |
| Other | 1 (3.3%) |
| **Patients dead at end follow-up** | 41 (11.1%) |
| *^1^* n (%) |  |

| **Table S6. Suggested biomarker groups by Cutoff Finder and STEPP functions** |  |
| --- | --- |
| **Biomarker groups with suggested cutoff***^1^* | **n = 371***^1^* |
| **HER2 copy number** |  |
| < 7.03 | 71 (19.1%) |
| ≥ 7.03 - < 14.03 | 247 (66.6%) |
| ≥ 14.03 | 53 (14.3%) |
| **HER2/CEP17** **ratio** |  |
| < 3.01 | 38 (10.2%) |
| ≥ 3.01 - < 11.32 | 320 (86.3%) |
| ≥ 11.32 | 13 (3.5%) |
| **ERBB2 mRNA** |  |
| < -1.05 | 52 (14.0%) |
| ≥ -1.05 - < 1.75 | 256 (69.0%) |
| ≥ 1.75 | 63 (17.0%) |
| *^1^* n (%); Suggested by Cutoff Finder and STEPP functions | |
